# A Veteran’s Manner of Life

**Published:** 1891-04

**Authors:** 


### A VETERAN'S MANNER OF LIFE.

A practical attempt to solve the question how to grow old, has been made recently by the Oesterreicher-Volksbildungs-Verein, which is composed of some of the most brilliant men and best thinkers in Austria, and has its headquarters in Vienna. For the edification of its members and the benefit of the public at large, the society sent a number of circulars to men of distinction who have obtained an advanced age, containing a series of questions in reference to their habits of life and the influences to which they attribute their health and vigor. It will cause, probably, little surprise to learn that one of the first replies which reached the club house was that of Count von Moltke. The circular, with his interesting answers to the interrogatories read as follows :

YOUTH.

Question—At what age did you begin to attend school, and how many hours a day did you study ?

Answer—In 1808, when eight years old. Four hours a day until 1810 ; after that year, ten hours daily.

Was your health, as a child, good or poor ?

Answer—Fair.

Did you pass your youth in the city or country ?

Answer—In the country until ten years old.

How many hours did you spend, as a rule, in the open air ?

Answer—Only a few hours, and no certain number.

Did you play athletic games and devote time to gymnastic exercises ?

Answer—Not as a rule.

How many hours did you sleep ?

Answer—Ten hours.

What general remarks do you care to make in regard to your youth?

Answer—It was unpleasant and unhappy, without sufficient nourishment, and was passed away from home.

MANHOOD.

Did you prepare yourself for your professiom in the city or in the country ?

Answer—In the city.

How many hours did you work each day ?

Answer—Different number at different times.

Do you ascribe to any habit, a particular influence upon your health ?

Answer—Temperance in all habits of life ; exercise in the open air whatever the state of the weather ; no day passed entirely within doors.

How long did you sleep ?

Answer—Between eight and nine hours.

What changes did you make after reaching an advanced age in your habits of life ?

Answer—None.

How many hours a day did you work in your fiftieth, sixtieth, seventieth and eightieth years?

Answer—It depended upon the demands of the times, and, therefore, often very many.

What has been your recreation?

Answer—Horseback riding, until I reached the age of eighty-six years.

How many hours do you now spend in the open air?

Answer—When on my estate in summer, half of the day.

How many hours do you sleep?

Answer—Still eight hours.

What peculiarities have you as to nourishment, etc?

Answer—I eat very little and make use of food extracts.

To what circumstances or conditions do you ascribe, in the main, your hearty old age?

Answer—To the grace of God and temperate habits of life.

COUNT VON MOLTKE, Field Marshal.

### FOOD AND CHARACTER.

Oliver Wendell Holmes, in a series of papers, both readable and valuable, which appeared in the columns of the *Atlantic*, gives his many friends among the lovers of good literature a great deal of sensible advice how they may healthfully and happily attain to a good old age; for what is length of days without health or happiness? Among other good things from the "Autocrat" of eighty years, we quote the following on the effects of diet upon character:—

Somebody has been writing to me about "oat-meal and literature," and somebody else wants to know whether I have found character influenced by diet; also whether, in my opinion, oat-meal is preferable to pie as an American national food.

In answer to these questions, I should say that I have my beliefs and prejudices; but if I were pressed hard for my proofs of their correctness, I should make but a poor show in the witness-box. Most assuredly I do believe that body and mind are much influenced by the kind of food habitually depended upon. I am persuaded that a too exclusively porcine diet gives a bristly character to the beard and hair, which is borrowed from the animal whose tissues these stiff-bearded compatriots of ours have too largely assimilated. I can never stray among the village people of our windy capes, without now and then coming upon a human being who looks as if he had been split, salted, and dried, like the salt fish which has built up his arid organism. If the body is modified by the food which nourishes it, the mind and character very certainly will be modified by it also. We know enough of their close connection to be sure of that without any statistical observations to prove it.

Do you really want to know "whether oat-meal is preferable to pie as an American national food"? I suppose the best answer I can give to your question is to tell you what is my own practice. Oat-meal in the morning, as an architect lays a bed of concrete to form a base for superstructure. Pie when I can get it; that is, of the genuine sort, for I am not patriotic enough to think very highly of the article named after the Father of his Country, who was first in war, first in peace—not first in pies, according to my standard.

